# Effect of Relative Marker Movement on the Calculation of the Foot Torsion Axis Using a Combined Cardan Angle and Helical Axis Approach

**DOI:** 10.1155/2012/368050

**Published:** 2012-05-14

**Authors:** Eveline S. Graf, Ian C. Wright, Darren J. Stefanyshyn

**Affiliations:** ^1^Human Performance Laboratory, Faculty of Kinesiology, University of Calgary, 2500 University Drive NW, Calgary, AB, Canada T2N 1N4; ^2^Adidas Innovation Team, Adidas, 5055 N Greeley Avenue, Portland, OR 97217, USA

## Abstract

The two main movements occurring between the forefoot and rearfoot segment of a human foot are flexion at the metatarsophalangeal joints and torsion in the midfoot. The location of the torsion axis within the foot is currently unknown. The purpose of this study was to develop a method based on Cardan angles and the finite helical axis approach to calculate the torsion axis without the effect of flexion. As the finite helical axis method is susceptible to error due to noise with small helical rotations, a minimal amount of rotation was defined in order to accurately determine the torsion axis location. Using simulation, the location of the axis based on data containing noise was compared to the axis location of data without noise with a one-sample *t*-test and Fisher's combined probability score. When using only data with helical rotation of seven degrees or more, the location of the torsion axis based on the data with noise was within 0.2 mm of the reference location. Therefore, the proposed method allowed an accurate calculation of the foot torsion axis location.

## 1. Introduction

Torsion of the foot is defined as the relative rotation between the forefoot and rearfoot about an axis parallel to the foot length axis (in the frontal plane) occurring mainly at the transverse talar and tarsometatarsal joint [1, 2]. This movement is especially important during athletic movements such as running, cutting, and landing after jumps because torsion allows the rearfoot to remain aligned with the shank thereby reducing the stress on the Achilles tendon and lowering the risk for ankle injuries such as sprains [2–4].

Studies looking at the effect of footwear on torsion showed a reduction in peak torsion angle by up to 15 degrees for individual subjects, potentially putting athletes at a higher risk of injury [2]. Consequently, the footwear industry has searched for solutions to reduce the shoe torsional stiffness in the midfoot area in order to allow for more natural motion. However, it was crucial to maintain the bending stiffness of the midfoot area to avoid midfoot flexion since shoes that allow this type of movement can force the foot into a nonphysiological position [2]. A torsion element that allowed for rotation of the foot about the longitudinal axis and also limited bending of the midfoot was developed [2]. However, the location of those torsion elements was not based on the anatomical rotation axis of the forefoot relative to the rearfoot because previous measurement techniques used to calculate torsion are based on Cardan angles or even simpler two-dimensional approaches, which do not allow for the calculation of the actual rotation axis.

The finite helical axis (FHA) method describes the motion of a rigid body as the rotation about and translation along a specific axis [5]. It can be used for a functional representation of joint movement as it has previously been done for the shoulder [6], the forearm/elbow [6–8], the knee [9, 10], and the ankle joint [11]. The FHA between forefoot and rearfoot, however, has not previously been examined but could lead to an identification of the torsional axis of the foot. An advantage of the FHA method over Cardan angles is that its results are only dependent on the definition of the reference coordinate system definition, while Cardan angles are dependent on both the reference and moving coordinate system [12]. One limitation of the FHA method, however, is that when the helical rotation is zero, the helical axis is undefined. This makes the FHA approach susceptible to error with small rotations [13]. While de Lange et al. (1990) discussed the use of data smoothing to increase the accuracy of the FHA parameters, other researchers excluded instances with small rotations from the finite helical axis calculation [14, 15]. Studies calculating the FHA of a single joint have shown that the axis location differed for different joint angles [9, 16]. It can therefore be assumed that the torsion axis as well will be moving over the course of the stance phase which makes it difficult for a shoe torsion bar to represent the floating foot torsion axis. It can be speculated that the location of the foot torsion axis during maximal torsion angles is of most interest when designing shoe torsion bars. Cases with small rotations, which are prone to error, should therefore not be included in the torsion axis calculation. Excluding small rotations is therefore an appropriate approach to increase the accuracy of the FHA calculation in this case.

When measuring torsion of the foot with conventional three-dimensional measurement techniques, markers are typically placed on the heel and the forefoot. The movement with the largest range of motion between those segments is flexion at the metatarsophalangeal joint. Consequently, calculating the FHA would not lead to a torsion axis, but to an axis that is mainly influenced by flexion. Thus in order to properly estimate the location of the torsion axis (the orientation is given as longitudinal to the foot), a new approach was needed. Therefore, the purposes of the present study were to (1) describe an approach that could accurately calculate the torsion axis of the foot using a modified finite helical axis approach that eliminates the effect of flexion at the metatarsophalangeal joint; (2) determine the sensitivity of the method to relative marker movement by systematically simulating marker movement; (3) determine the minimal helical rotation needed to achieve an acceptable low level of error caused by the simulated relative marker movement.

## 2. Methods

**Figure 1:**
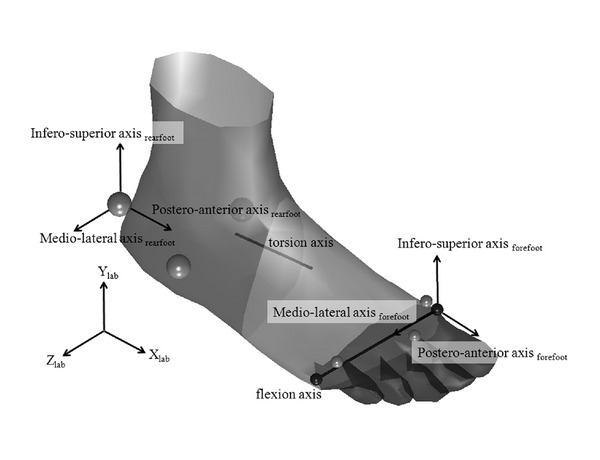
Foot model with (1) segment markers (light gray) and metatarsophalangeal joint markers (dark gray) (2) rearfoot and forefoot coordinate systems and (3) torsion and flexion axes.

A modified helical axis approach was developed using a combination of Cardan angles and the finite helical axis method to determine the torsion axis during movements based on a kinematic foot model with a forefoot and a rearfoot segment. This foot model consisted of three markers on the heel and three markers on the forefoot (Figure 1).

Additional markers were added during a neutral trial (upright standing with the foot visually aligned with the *x*-axis of the lab coordinate system) defining the metatarsophalangeal joint with a marker on each of the first and the fifth metatarsal heads. The rearfoot segment coordinate system (RCS) was oriented parallel to the orthogonal, right-handed lab coordinate system (LCS) with the origin at the central heel marker. The segment coordinate system of the forefoot (FCS) originated at the marker positioned at the first metatarsal head. The mediolateral axis (flexion) pointed towards the lateral MTP marker. The posteroanterior axis (inversion) was in the same plane as the posteroanterior axis of the rearfoot coordinate system. The inferosuperior axis (adduction) was calculated as the cross product between the mediolateral and the posteroanterior axes (Figure 1). During movements, the mediolateral axis of the FCS was assumed as the forefoot flexion axis. The transformation (rotation and translation) of the rearfoot relative to the forefoot was calculated using the singular value decomposition algorithm described by Soderkvist and Wedin [17]. To determine the amount of flexion occurring at the metatarsophalangeal joint, Cardan angles were calculated with the order of rotation being first around the mediolateral axis, second around the inferosuperior axis, and third about the posteroanterior axis. Each rotation was performed about an axis of the moving coordinate system. The rotation about the mediolateral flexion axis was removed from the relative movement between forefoot and rearfoot by multiplying the inverse of the matrix representing the flexion angle with the total rotation matrix. Based on the resultant transformation matrix, the finite helical axis parameters (helical angle, orientation, and location) were calculated [5].

**Figure 2:**
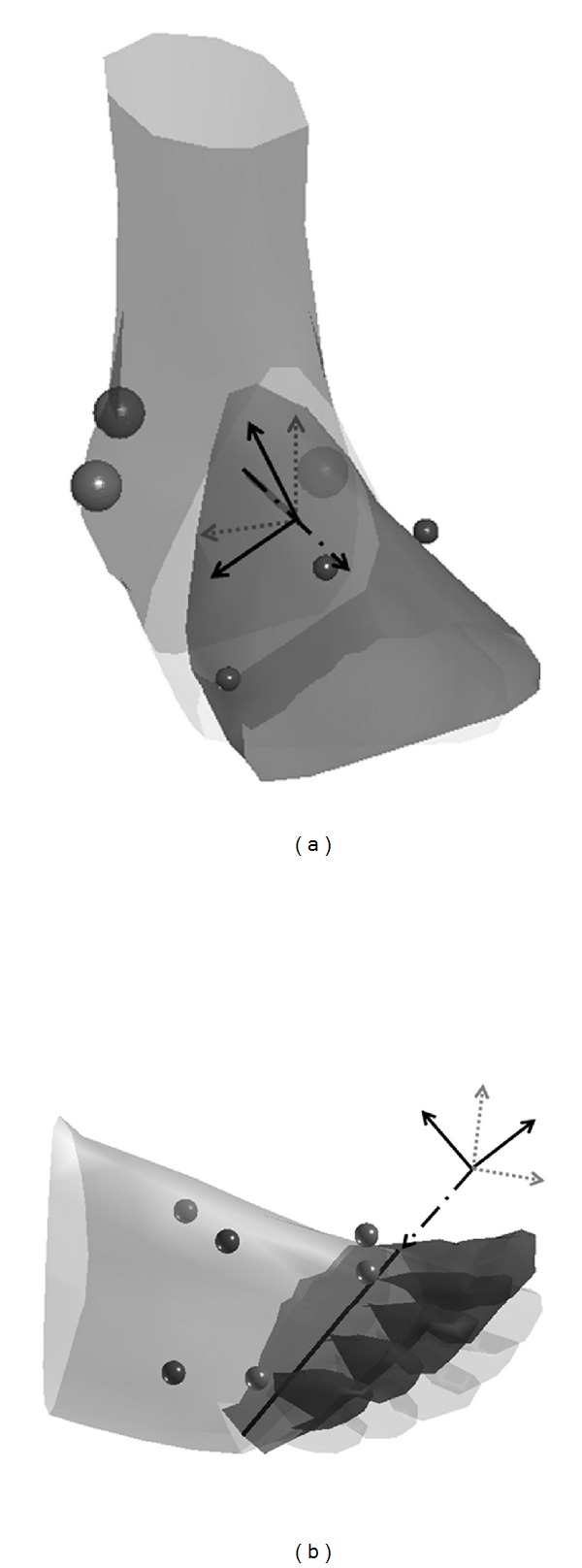
Modeling of foot torsion and flexion with dashed lines representing distal segment coordinate system in neutral position and solid lines representing distal segment coordinate system after rotation around dash-dot line: (a) rotation of midfoot relative to rearfoot (torsion) and (b) rotation of forefoot relative to midfoot (flexion).

The validation of the modified helical axis method was based on a simulation of a three-segment foot (fore-, mid-, and rearfoot). Torsion has been described as a movement occurring mainly at the transverse tarsal and tarsometatarsal joints [1], which can be simplified as a movement between the rearfoot and the midfoot. Flexion, however, occurs primarily between the midfoot and the forefoot segment. Therefore, in order to functionally represent foot movement, a foot model based on three segments was chosen for the simulation. Matlab software (Version 7.5, The MathWorks, MA, USA) was used for developing the model and the analysis. Three markers were used to describe the midfoot movement (Figure 2). The midfoot segment coordinate system (MCS) was defined as parallel to the LCS with the origin at the central midfoot marker. For the forefoot and the rearfoot, the same marker setup and coordinate system definitions were used as previously described. Foot torsion was simulated as a rotation between the rearfoot and midfoot about an axis parallel to the posteroanterior RCS axis (Figure 2(a)). Flexion between the midfoot and forefoot segments occurred around the FCS mediolateral axis (Figure 2(b)). Cardan rotations about the torsion and flexion axes were used as input variables. The torsion angle ranged between 1 and 15 degrees, and the flexion angle ranged between 1 and 20 degrees (both with 1-degree increments) resulting in 300 different samples. The maximal rotation angles represented the peak angles that can be observed in athletic cutting movements [18]. The outputs of the modified helical axis calculation were the helical rotation angle, the location of the helical axis relative to the rearfoot coordinate system, and the orientation of the helical axis. The posteroanterior location of the helical axis was fixed at 200 mm. This location approximates the posteroanterior center of the midfoot of a size US13 foot. For the determination of the torsion axis, only the location of the helical axis was considered because the orientation of the torsion axis is predetermined as perpendicular to the frontal plane.

In order to determine the effect of relative marker movement on the torsion axis calculation, error in the marker position was simulated by adding a random value between −5 and 5 mm to each of the three coordinates defining the marker position of the forefoot markers. Calculating the rotation matrix according to Soderkvist and Wedin [17] based on marker data allowed calculating the residual matrix which contains the difference between the calculated and the actual marker position. Based on this residual matrix, the root mean square (mean residual) of the individual entries was calculated which is an indication of the amount of error in the rigid-body assumption. Using a random error of −5 to 5 mm leads to similar mean residuals as observed in pilot data during movements like walking or cutting when skin markers are attached to the bare foot. During the pilot testing using 2 subjects, the marker placement on the forefoot and rearfoot and the segment coordinate system definitions were chosen according to the setup described previously and, therefore, represented the testing situation used in the simulation. Using the modified helical axis approach, the location of the torsion axis and the helical angle were calculated. The results of the location calculation were compared to the data without error for all 300 samples. The differences between these data were assessed using a one-sample *t*-test with a significance level of 5%.

Since the FHA method is sensitive to measurement errors when rotations are small [13], samples (one of the 300 specific combinations of flexion and torsion angle) with very small rotations needed to be excluded from the modified helical axis analysis to minimize the error. However, when discarding too many samples, the error in the helical axis calculation would increase again because too few data points would be available. Therefore, a threshold angle, representing the helical rotation that provided the most accurate results, needed to be determined. In order to determine this threshold angle, different subsets of data were created based on the amount of helical rotation. For the subsets, samples with a helical rotation of less than a specified cut-off angle were excluded; the cut-off angle was systematically increased from 0 degree (no exclusion) to 10 degrees with 1-degree increments.

The average location in both the inferosuperior and mediolateral directions of the data subsets was compared by one-sample *t*-test to the average location of the data that did not contain any error to test if they were significantly different from each other. The *P* values of the two location variables were combined using the Fisher combined probability test [19] and the cut-off angle that led to the highest combined *P* value which was selected as the threshold.

## 3. Results

The largest difference between the data containing error and the data with no error for both location variables of the torsion axis occurred when no cases were excluded (Table 1). When small angles were excluded, the difference between the data with and without error decreased. Table 1 also displays the individual and combined *P* values for all cut-off angles. The maximal combined *P* value, indicating the smallest difference between the data with and without error, occurred at a cut-off of 7 degrees. The difference between data with error and the reference data was less than 0.2 mm for both location directions. The mean residual for the forefoot markers, where the error was introduced, was 2.7 mm with a range of 0.7 mm to 5.4 mm.

## 4. Discussion

The purpose of this study was to develop an approach to calculate the torsion axis of the foot, to determine the effect of nonrigid segments on the results of a modified helical axis approach, and to find the minimal amount of rotation around the finite helical axis necessary to obtain accurate results. The finite helical axis method has been used to determine rotation axes of joints during movements [6–12]; however, it is susceptible to errors caused by the nonrigidity of segments and noise in the landmark coordinates especially with small angles of rotation [13, 16]. The method described here was a combination of the finite helical axis approach and Cardan angles to calculate the location of the rotation axis between the forefoot and the rearfoot without the influence of forefoot flexion. The results of this study indicate that the described approach is susceptible to errors due to violation of the rigid-body assumption; however, after exclusion of cases with small rotation, the error is reduced, and therefore, the results increase in accuracy.

Comparing the data containing error with the reference data containing no error showed that there can be large discrepancies between the two conditions, especially in the vertical location of the axis, which differs by about 20 mm. This indicates that errors in landmark coordinates can have large effects on the results, even though the results were not significantly different; a finding that is in agreement with previous studies [10, 13, 16]. The error introduced to the marker position was random noise. During a dynamic movement, relative marker movement does not occur randomly but is dependent on factors like acceleration and joint excursion which has not been taken into consideration for the described model. However, a study comparing bone-anchored markers to markers place on the skin failed to find systematic differences between the two approaches [20]. This makes it difficult to introduce error to the data that mimics the violation in the rigid body assumption observed in dynamic data.

It is known that using more than three markers per segment increases the accuracy of the calculation of segment orientation [21]. It can, therefore, be argued that when using more markers on the forefoot, the torsion axis location could be calculated with higher accuracy. However, the forefoot is very limited in space, and it would be very difficult to attach more than three markers.

When removing cases where the rotation about the helical axis is small, the error became smaller. The goal was to find the threshold angle that leads to the largest average *P* value for the two output variables when comparing the data containing errors with the data that did not contain any error. The results showed that a threshold angle of seven degrees leads to the highest combined *P* value (Table 1) which indicates that cases with helical rotations of less than seven degrees should be excluded from the analysis to reduce the error in the helical axis calculation due to nonrigid bodies. This threshold angle, however, depends on the range of torsion occurring. For a dynamic movement where only small amounts of midfoot rotation are occurring, a threshold of 7 degrees could be too high because it would exclude too many cases in order to still accurately calculate the helical axis. The values used in this study (torsion from 1 to 15 degrees and flexion form 1 to 20 degrees) represent the average torsion range observable during cutting movements [18]. Therefore, the threshold of 7 degrees should not lead to exclusion of too many cases for studies focusing on similar movements. Table 1 also shows that while a cut-off of seven degrees leads to the highest *P* value, cut-offs of six, eight, and nine degrees also resulted in combined *P* values of over 0.90 for the axis locations. However, those are also high or even higher cut-offs which will not change the limitation discussed above. The next highest *P* value is then at the cut-off of one degree (*P* = 0.89). That shows that when the finite helical axis or torsion axis location needs to be calculated of a movement with a small range of motion, cases with rotations of less than one degree should be excluded to increase the accuracy of the result.

Future research projects should apply the described method to kinematic data of athletic movements in order to determine the torsion axis location of the human foot. Knowledge of the foot torsion axis location would allow locating a shoe torsion element in a way so that the shoe torsion axis location coincides with the foot axis. It remains to be determined if such a shoe design changes the movement of the foot in the shoe. It has been shown that footwear with high torsional stiffness tends to increase the risk of lateral ankle sprains [22]. It is, however, unknown if the torsion axis location of the shoe has any influence on injury risk. Consequently, more research focusing on the influence of foot and footwear torsion and torsion axis location on lower extremity injuries is needed.

## 5. Conclusions

The purpose of this study was to describe an approach that can be used to calculate the torsion axis location of the foot. Further, the sensitivity of the method to relative marker movement was assessed using a simulation approach, and the minimal rotation needed for accurate results was determined. The described modified finite helical axis approach allows the calculation of the rotation axis between the forefoot and the rearfoot without the influence of forefoot flexion at the metatarsophalangeal joint. It allows the calculation of the torsion axis location of the foot during movements. This information could be used to develop functional torsion elements in shoes that allow a more natural movement of the foot with the shoe.

## Figures and Tables

**Table 1:** Average and standard deviation of the torsion axis location in the inferosuperior and mediolateral directions for the data without and with error (and different cut-off angles); *P* value of comparison to data without error and combined *P* value of location in *y* and *z* (based on Fisher's combined probability); (number) in first column represents amount of samples included in analysis.

	Location in inferosuperior direction [mm]	Location in mediolateral direction [mm]	Fisher's combined probability
No error	−25.45 ± 0.00	3.05 ± 0.00	
Cut-off 0° (300)	−45.10 ± 320.03	7.70 ± 66.98	
*P* value	0.29	0.23	0.25
Cut-off 1° (296)	−29.59 ± 134.83	3.22 ± 45.50	
*P* value	0.60	0.95	0.89
Cut-off 2° (282)	−23.41 ± 61.24	4.81 ± 32.66	
*P* value	0.58	0.37	0.54
Cut-off 3° (263)	−26.18 ± 45.00	4.24 ± 28.41	
*P* value	0.79	0.50	0.76
Cut-off 4° (244)	−24.41 ± 43.49	5.41 ± 25.47	
*P* value	0.71	0.15	0.34
Cut-off 5° (224)	−25.19 ± 13.69	4.22 ± 23.16	
*P* value	0.78	0.45	0.72
Cut-off 6° (197)	−25.28 ± 11.49	3.48 ± 19.55	
*P* value	0.83	0.76	0.92
Cut-off 7° (179)	−25.60 ± 10.14	2.94 ± 18.44	
*P* value	0.84	0.94	0.98
Cut-off 8° (162)	−25.60 ± 9.54	3.45 ± 17.39	
*P* value	0.84	0.77	0.93
Cut-off 9° (139)	−25.47 ± 9.45	2.40 ± 16.08	
*P* value	0.98	0.63	0.92
Cut-off 10° (119)	−25.17 ± 8.16	1.09 ± 15.73	
*P* value	0.71	0.18	0.38

## References

[1] Debrunner H (1985). *Biomechanics of the Foot*.

[2] Segesser B, Stüssi E, von A Stacoff M, Kälin X, Ackermann R (1989). Torsion—a new concept in construction of sports shoes. Motion excursion of the foot in athletic stress—anatomical and biomechanical observations and their effects on construction of sports shoes. *Sportverletzung Sportschaden*.

[3] Stacoff A, Kaelin X, Stuessi E, Segesser B (1990). [Foot Movement during Landing after a Jump]. *Zeitschrift fuer Orthopaedie*.

[4] Stacoff A, Kaelin X, Stuessi E, Segesser B (1989). The torsion of the foot in running. *International Journal of Sport Biomechanics*.

[5] Spoor CW, Veldpaus FE (1980). Rigid body motion calculated from spatial co-ordinates of markers. *Journal of Biomechanics*.

[6] Cattrysse E, Baeyens J-P, van Roy P, Van de Wiele O, Roosens T, Clarys JP (2005). Intra-articular kinematics of the upper limb joints: a six degrees of freedom study of coupled motions. *Ergonomics*.

[7] Tay SC, van Riet R, Kazunari T, Amrami KK, An KN, Berger RA (2010). In-vivo kinematic analysis of forearm rotation using helical axis analysis. *Clinical Biomechanics*.

[8] Chin A, Lloyd D, Alderson J, Elliott B, Mills P (2010). A marker-based mean finite helical axis model to determine elbow rotation axes and kinematics *in vivo*. *Journal of Applied Biomechanics*.

[9] van den Bogert AJ, Reinschmidt C, Lundberg A (2008). Helical axes of skeletal knee joint motion during running. *Journal of Biomechanics*.

[10] Blankevoort L, Huiskes R, de Lange A (1990). Helical axes of passive knee joint motions. *Journal of Biomechanics*.

[11] Tuijthof GJM, Zengerink M, Beimers L (2009). Determination of consistent patterns of range of motion in the ankle joint with a computed tomography stress-test. *Clinical Biomechanics*.

[12] Shiavi R, Limbird T, Frazer M, Stivers K, Strauss A, Abramovitz J (1987). Helical motion analysis of the knee—I. Methodology for studying kinematics during locomotion. *Journal of Biomechanics*.

[13] Woltring HJ, Huiskes R, De Lange A (1985). Finite centroid and helical axis estimation from noisy landmark measurements in the study of human joint kinematics. *Journal of Biomechanics*.

[14] Gallo LM, Airoldi GB, Airoldi RL, Palla S (1997). Description of mandibular finite helical axis pathways in asymptomatic subjects. *Journal of Dental Research*.

[15] Hayashi K, Tanaka H, Hikita K, Mizoguchi I (2004). Basic behavior of the finite helical axis in a simple tooth movement simulation. *Medical Engineering and Physics*.

[16] de Lange A, Huiskes R, Kauer JMG (1990). Effects of data smoothing on the reconstruction of helical axis parameters in human joint kinematics. *Journal of Biomechanical Engineering*.

[17] Soderkkvist I, Wedin PA (1993). Determining the movements of the skeleton using well-configured markers. *Journal of Biomechanics*.

[18] Davis EM, Landry SC, Nigg BM (2009). Torsion of the foot in low cut basketball shoes in four cutting movements. *Footwear Science*.

[19] Fisher RA (1958). *Statistical Methods for Research Workers*.

[20] Nester C, Jones RK, Liu A (2007). Foot kinematics during walking measured using bone and surface mounted markers. *Journal of Biomechanics*.

[21] Challis JH (1995). A procedure for determining rigid body transformation parameters. *Journal of Biomechanics*.

[22] Graumann L, Walther M, Krabbe B, Kleindienst F (2007). [The influence of sports-shoes mechanical properties on the frequency of lower-extremity athletic injuries]. *Sportorthopaedie Sporttraumatologie*.

